# Dating the beginning of the Roman viticultural model in the Western Mediterranean: The case study of Chianti (Central Italy)

**DOI:** 10.1371/journal.pone.0186298

**Published:** 2017-11-15

**Authors:** Riccardo Aversano, Boris Basile, Mauro Paolo Buonincontri, Francesca Carucci, Domenico Carputo, Luigi Frusciante, Gaetano Di Pasquale

**Affiliations:** Department of Agricultural Sciences, University of Naples Federico II, Portici, Italy; Universita degli Studi di Siena, ITALY

## Abstract

Although domestication of the grapevine (*Vitis vinifera* L.) has been extensively documented, the history of genotype selection and evolution of vineyard management remain relatively neglected fields of study. The find of 454 waterlogged grapevine pips from a well-dated Etrusco-Roman site in the Chianti district (Tuscany, Central Italy) is an extraordinary chance to gain insights into the progress of viticulture occurring in a key historical period in one of the world's most famous wine regions. The molecular and geometrical analyses of grape seeds showed (a) the presence in the site of different grapevine individuals and (b) a sudden increase in pip size, occurring at around 200 BC, whic explainable by the selection and introduction of new varieties. In this period, the Etruscans settlers in Chianti were stimulated by northward-expanding Roman culture to use novel vineyard management practices. We hypothesize that one of the most important innovations may have been the introduction of pruning, inducing vine physiological conditions more favorable to pip growth. Such changes were the consequence of specific entrepreneurial choices made by the Romans in a period of economic investment in grape cultivation and wine making to satisfy the increased trade demand after the conquest of the Central-Western Mediterranean basin.

## Introduction

Domestication of the cultivated grapevine (*Vitis vinifera* L.) is traditionally regarded as first taking place in the Caucasus, an area which shows high genetic diversity for this crop [1]. It spread to Egypt and Mesopotamia and then throughout the Mediterranean area. Molecular analysis shows the multi-geographic contribution of wild grapevine to the regional gene pools of cultivated varieties, suggesting independent secondary domestication sites in the western Mediterranean [2]. Italy is one of the countries where this crop has been traditionally grown for millennia. Here, archaeobotanical finds suggest an increasing trend of cultivation from the 9^th^ to the 7^th^ century BC [3]. Under Roman influence, intensive viticulture was also introduced to much of Europe's temperate regions, most notably to France and Germany [4]. Research on chloroplast DNA polymorphisms has revealed that several Italian grapevine cultivars are highly related to the Near-Eastern wild grape group [5] and compatible with the hypothesis of local domestication events or interbreeding with wild grapes growing in the same area [6,7].

Despite the relative richness of multidisciplinary data and the advent of new archaeological and genetic techniques, the patterns and processes of grapevine domestication, diversification and technological innovations are still widely discussed [8]. Archaeobotanical evidence can help us to shed light on such processes. In this regard, the remains of wild and domesticated grape pips can be particularly useful. With their morphology and structure specifically designed to store genetic information, seeds are a promising source of material to investigate the history of grapevine cultivation and the wine trade [9]. Morphological analysis of ancient grape pips has long been used to distinguish wild and cultivated subspecies [10–13]. Recently, on the basis of a sub-regional reference sample of modern wild and cultivated grape pips, others have proposed, as an alternative tool, the functional analysis of grapevine seed outlines [14,15], while Bouby et al. [16] implemented traditional morphometric measurements with cluster and multivariate statistical analyses. Providing accurate criteria discriminating *V*. *vinifera* subspecies, well-preserved archaeological waterlogged pips have been compared, showing the changes in traits in relation to the domestication process and suggesting starting points on the history of grapevine cultivars [14–16].

Morphological analysis can be combined with additional techniques. In particular, the analysis of ancient DNA (aDNA) recovered from archaeological plant residues has made important advances in recent years [17]. It can help shape our understanding of past grapevine diffusion and the genetic changes that have occurred in domesticated populations [1,9,18–20].

Grapevine domestication trajectories are being increasingly documented [21]. However, the timing and the pace governing improvements in winegrowing and enhancement of the characteristics of cultivated grapes remain to be elucidated. The find of waterlogged grape pips from a well-dated Etrusco-Roman archaeological site in the core of the Chianti district (Tuscany, Central Italy), one of the oldest and most renowned wine regions in Europe, constitutes an extraordinary chance to shed light on the evolution of viticulture during the Etruscan and Roman periods in the heart of the Italian peninsula. This study combines a geometrical analysis of grape seed morphology with a molecular approach: 1) to highlight changes that occurred between the Etruscan and Roman Ages (from the 3^rd^ century BC to the 1^st^ century AD), a key period in the history of viticulture; 2) to interpret pip shape diversity in relation to the supposed changes in agronomic techniques; and 3) to attempt genetic affiliations between ancient samples and modern varieties.

## Materials and methods

### Archaeological background

The Etruscan civilization spread between the 9^th^ and 8^th^ centuries BC primarily along Italy's upper Tyrrhenian seaboard, so-called Etruria. Here, viticulture for wine making became an important economic activity for the first time [22,23]. According to historical sources, Etruscans trained grapevines up live trees (so-called *lambruscaia*), exploiting their characteristic as climbing plants [24–26].

**Fig 1:**
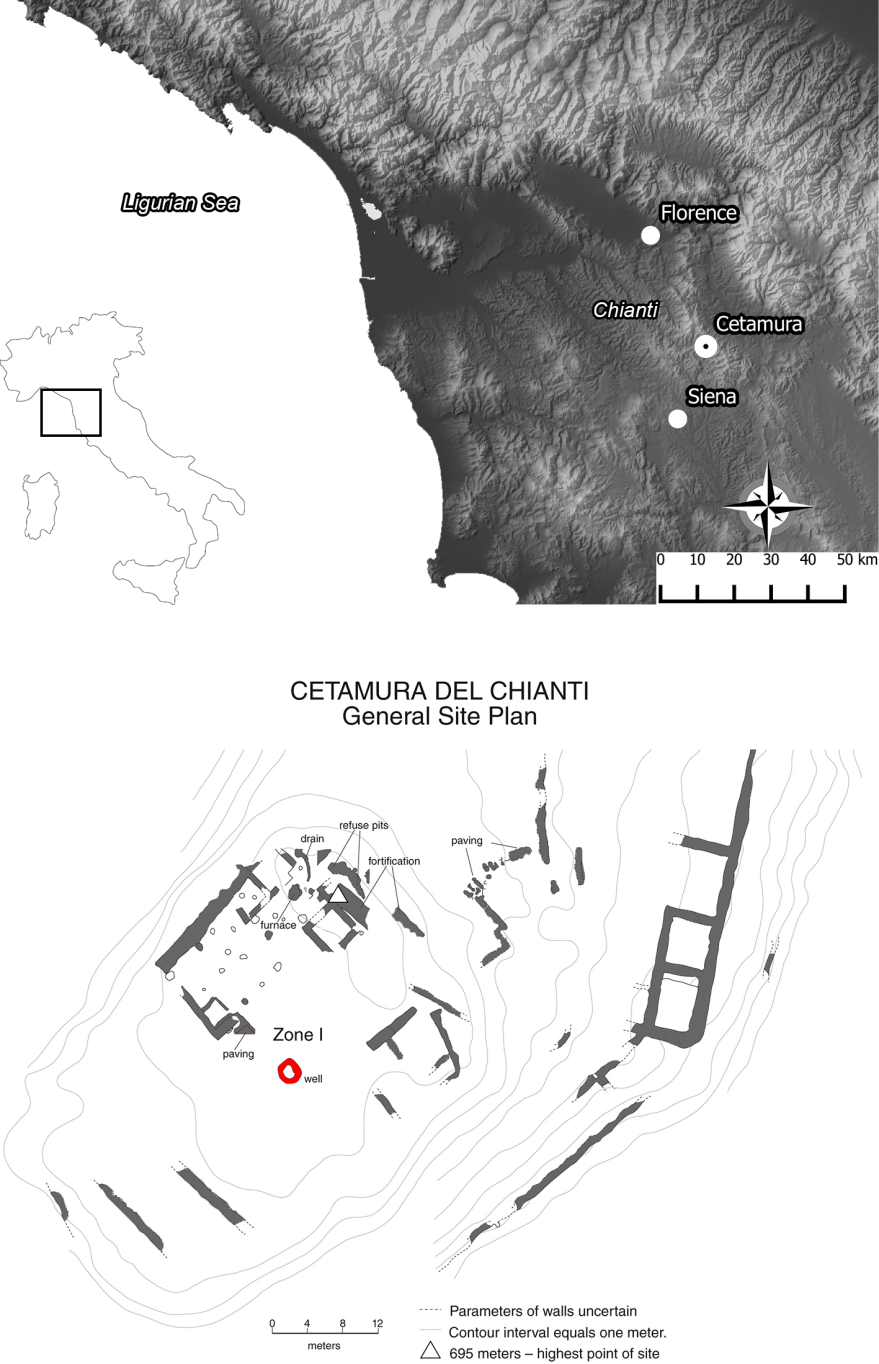
The study context: Location of the Chianti and Cetamura site in central Italy (from http://wms.pcn.minambiente.it/ogc?map=/ms_ogc/WMS_v1.3/raster/DTM_20M.map, under a CC BY license, with permission from ministry of the environment and protection of land and sea—National geoportal, original copyright 2001). General site plan of Cetamura (from [31] for illustrative purposes only).

The present-day hill region of Chianti was an important area of inland northern Etruria (Fig 1), inhabited between the 7^th^ and 5^th^ centuries BC and later from the 3^rd^ century BC [27,28]. While from the 4^th^ century BC southern Etruria was gradually conquered by the Romans, Chianti and the nearby cities in the interior of northern Etruria preserved the archaic Etruscan culture until ca 200 BC when, under the weight of Roman pressure and influence, the economic, political, and social traditions gradually changed [29]. This long presence of the Etruscan culture ended definitively in the mid-1st century BC after a period of devastating power struggles on the Italian mainland.

The archaeological settlement of Cetamura in Chianti is located at an altitude of 684 m a.s.l. (Fig 1). The Etruscans inhabited this site from the end of the 4^th^ century BC to the very last years of the Late Etruscan Age, ca 150–100 BC, whereas the subsequent Roman presence lasted until the 2^nd^ century AD [30].

### Archaeobotanical sampling

**Fig 2:**
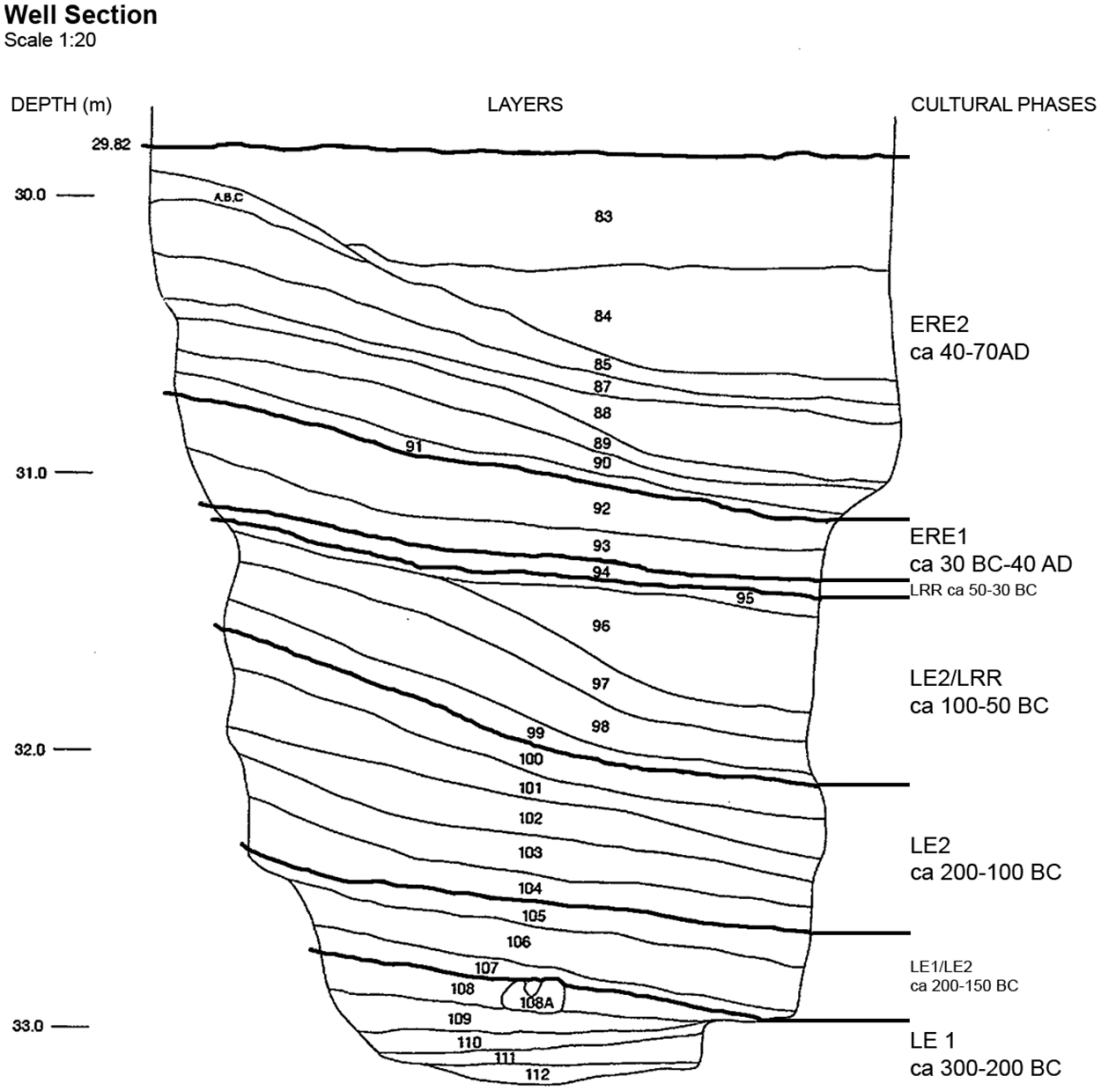
Well section with archaeological layers, depth (in meters) and cultural phases (from [32] for illustrative purposes only).

In Cetamura, the excavation of a rock-cut well was carried out from 2011 to 2014. The well is 33 m deep and dates from 300 BC (Fig 2). Seven chronological phases were detected, suggesting use for around 370 years [32]. The wet conditions of the well favored the excellent preservation of several botanical remains including leaves, wood, seeds and fruits. Sediment samples for archaeobotanical analysis were taken from 29 archaeological layers at the site, between 29.82 m and 33.42 m in depth (Fig 2). The samples were labeled and dated according to the layer, and then processed with an ‘‘Ankara” type flotation machine equipped with a 0.5 mm mesh in the floating tank. Floated-out botanical remains were recovered from mesh sizes 4, 2, 1, 0.50 and 0.25 mm.

A total of 454 waterlogged grape pips were recorded from 21 archaeological layers, covering the following five cultural phases: Late Etruscan 1 (LE1, 300–200 BC), Late Etruscan 2 (LE2, 200–100 BC), Late Roman Republic (LE2/LRR, 100–50 BC), Early Roman Empire (ERE1, 30 BC-40 AD), Early Roman Empire 2 (ERE2, 40–70 AD) (Table 1). Twenty-two pips were used to extract DNA for molecular analysis, whereas all 454 pips were subjected to biometric determinations. No permits were required for the molecular and geometrical analyses performed in this study, which complied with all relevant regulations.

**Table 1:** Absolute values of the pips from the Cetamura well grouped by cultural phases and archaeological layers.

Cultural phase		Layers	Pips		
			Intact	Damaged	Total
Early Roman Empire 2	ERE2	83	65	9	
		84	5	4	
		85	24	2	
		86	81	17	
		87	31	2	
		88	24	7	
		90	8	1	
		91	42	8	
			280	50	330
Early Roman Empire 1	ERE1	92	15	2	
		93	8	2	
			23	4	27
Transition Late Etruscan 2 and Late Roman Republican	LE2/LRR	96	4		
		97	6		
		98	4		
		99	2		
			16		16
Late Etruscan 2	LE2	100	7		
		101	29	1	
		102	21		
		103	1		
			58	1	59
Late Etruscan 1	LE1	110	8		
		111	3		
		112	9	2	
			20	2	22
			397	57	454

Intact and discarded pips were reported according to biometric analysis.

### Molecular analyses

The experimental procedures described in this work were conducted in a physically separate workplace dedicated to aDNA, which had never been used for isolation of contemporary grapevine nucleic acids. All necessary precautions to avoid aDNA contamination were adopted as described in S1 Methods. Twenty-two ancient grape pips belonging to different phases were used for DNA extraction. Due to the limited number of samples excavated from the same layer, single-seed extraction was carried out in order to capture the genetic signature of each sample rather than a mixed signal from multiple individuals. DNA from ancient grape pips was extracted using the ChargeSwitch Forensic DNA Purification Kit (Life Technologies, Carlsbad, CA, USA). Positive controls were avoided to circumvent contamination risk, while negative controls were always performed. Extracted DNA was diluted to a concentration of 0.1 ng/uL and stored in Eppendorf at -20°C. Microsatellite analysis was carried out with 23 nuclear and 11 chloroplast markers as reported in S1 Table. We included the nine SSR loci (VVS2, VVMD5, VVMD7, VVMD25, VVMD27, VVMD28, VVMD32, VrZAG62, and VrZAG79) that the European scientific community selected and chose for grapevine identification, standardization and exchange of information [33]. All the PCR amplifications were repeated at least three times using different thermocyclers situated in separate laboratories with different research teams. PCR reactions, amplicon separations and data analysis were carried out as reported in Villano et al. [34] with some modifications (S1 Methods). SSR profiles previously obtained were finally integrated with 60 further profiles from as many genotypes [34]. For markers that gave amplicons in at least one pip per phase, data were scored for the presence or absence of each allele in all genotypes, and a genetic distance matrix was calculated using Dice’s coefficient [35,36]. A dendrogram was built through the UPGMA (unweighted pair group method with arithmetic mean) method using R software, version 3.2.1 (2015-06-18).

### Biometric analysis

Each intact pip was individually photographed with an Olympus DP20 digital camera connected to an Olympus SZX7 stereomicroscope equipped with an 8x magnification lens. For each pip we measured four morphological parameters: pip length, pip breadth, stalk length and chalaza position [16]. Since pip size has been reported to vary greatly among different grape varieties and to be correlated to berry size [37], we also measured pip surface area and pip perimeter as integrated measures of pip size. Stalk length and chalaza position were measured manually with ImageJ 1.40 software [38] using a graded (1 mm grid) paper to calibrate distances. The other measurements were carried out automatically using Tomato Analyzer 3.0 software [39]. The Stummer shape index was calculated for each pip as the ratio of pip breadth to pip length [13].

The significance of the differences between phases in pip biometric features and in the calculated indexes was assessed by one-way ANOVA using the Duncan test (p ≤ 0.05) as a post-hoc test for separation of means. A discriminant analysis procedure was applied to the experimental data to distinguish among pips of the different cultural phases using simultaneously all the measured biometric parameters. All the statistical analyses were performed using SPSS software package (SPSS Inc., Chicago, Illinois, U.S.A.).

## Results

### Molecular analysis

**Fig 3:**
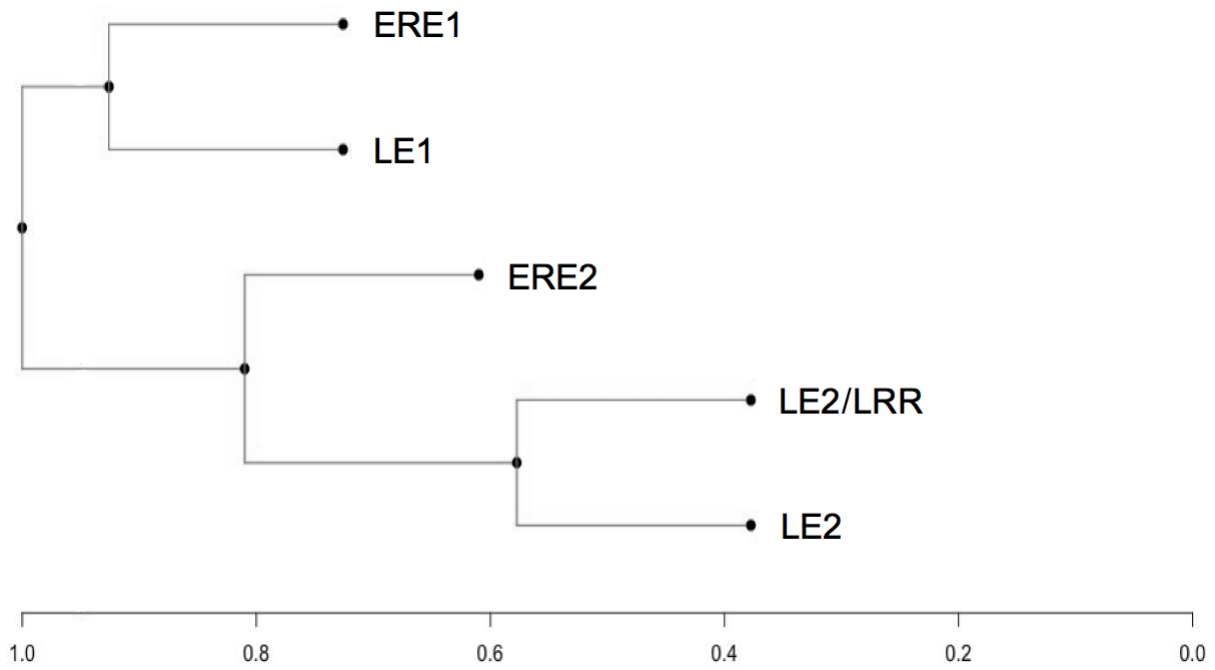
SSR-based UPGMA (unweighted pair group method with arithmetic mean) dendrogram depicting the genetic relationship among different ancient grape pips. Genetic distances were evaluated using Dice’s coefficient.

aDNA was successfully isolated from 15 ancient pips out of 22 initially used. Microsatellite amplifications were carried out with 34 SSR markers, but only 14 gave amplicons in 15 ancient pips belonging to the different cultural periods as follows: 1 to ERE1, 2 to LE1, LE2 and LE2/LRR and 8 to ERE2. Details on the positive and negative amplifications obtained are reported in Table 2. Overall, within the same sample, cytoplasmic DNA (cpDNA) was amplified more often than single-copy nuclear DNA (nuDNA) sequences. This is likely due to DNA degradation, as organellar genomes are found in multiple copies per living cell, increasing their chances of out-surviving those of rarer single-copy nuDNA sequences. Six loci (VrZag47, VrZag112, CCMP2, CCMP3, CCMP6 and CCMP7) were successfully amplified in at least one pip per phase, while the others allowed amplification in only some of the five phases taken into consideration. Overall, 45 alleles were identified and their sizes ranged from 74 bp (CCMP8) to 286 bp (ccSSR5) (Table 2). No genetic differences were found within the samples of the same cultural period, except for ERE2 pips, which displayed polymorphisms at six loci. Nuclear SSR (nuSSR) appeared to be homozygotes, which is consistent with grape hermaphroditism and its consequent propensity for selfing. However, several apparent homozygotes are likely to be heterozygotes with one amplified and one null allele. In addition, undetected heterozygosis due to allele drop after degradation of the ancient genetic material cannot be excluded. To better appreciate the relationships among pip samples, a UPGMA dendrogram was built (Fig 3) using those markers that were successfully amplified in at least one pip per age. According to genetic distances two main groups were distinguished. Cluster analysis placed the Late Etruscan 1 and Early Roman Empire 1 samples next to each other. The remaining ancient samples (LE2, LE2/LRR and ERE2) fell into the second group.

**Table 2:** Microsatellite profiles found in ancient pips belonging to five different cultural periods.

Microsatellite locus	LE1 (no. pips: 2)	LE2 (no. pips: 2)	LE2/LRR (no. pips: 2)	ERE1 (no. pips: 1)	ERE2 (no. pips: 8)
VrZAG47	182	184	108, 154	166	180
VrZAG112	254	244, 254	244, 254	250, 254	244, 254
VVS2	-	110, 114	-	110, 114	130, 142
VVMD7	-	-	-	218	267 [259]
CCMP1	-	156	156	-	156
CCMP2	204	205	205	207	205 [206]
CCMP3	128	189	130	204	130
CCMP6	110	111	111	128	111 [120]
CCMP7	123	120	120	125	148
CCMP8	-	74	74	87	75
ccSSR5	-	286	273	-	274 [281]
CCMP5	-	-	-	-	120 [121] [135]
CCSSR14	-	220	-	-	221
VVS5	-	132	-	-	131

For each locus, the allele size detected in ancient pips is reported as the length in base pair (bp). Heterozygous sites are shown as two alleles of different length. Allelic variants found in pips belonging to the same cultural period are reported in square brackets. Undetected alleles are denoted with a dash (-).

In order to attempt a comparison of ancient samples with the modern accessions mainly cultivated in Italy, we used the database owned by our research group, which includes the SSR profiles of about 100 modern accessions [34]. We focused on the detection of the current accessions which still preserved the ancient allelic variants in their genomes and could therefore be evolutionarily connected to the ancient samples (Table 3). The locus VrZAG47 presented the 154, 166 and 182 allelic variants in 2, 9 and 1 accessions, respectively. VrZAG112 shared the 244 allelic variant with Lambrusco maestri and *V*. *labrusca* L., while both 250 and 254 alleles were detected in the Barbera accession. Similarly, at least one of the two VVS2 allelic variants (130 and 142) was conserved in 12 modern accessions, while both isoforms were present in 8 accessions. CCMP2 locus shared the 107 allele with many *V*. *vinifera* accessions. The alleles detected at the other loci were never identified in the database.

**Table 3:** Detected allelic variants of ancient samples still conserved in 122 modern cultivars.

Microsatellite locus	Detected allelic variant	Cultural period	Found in
VrZAG47	154	LE2/LRR	Curniciello, Montonico
	166	ERE1	Guarnaccia, Lambrusco Salamini, Lambrusco maestri, Montepulciano, Pellecchiona, Sommarello, Livella, Merlot, Sirica
	182	LE1	Catalanesca
VrZAG112	244	LE2, LE2/LRR, ERE2	Lambrusco maestri and *Vitis labrusca*
	250	ERE1	Barbera
	254	LE1, ERE2	Barbera
VVS2	130	ERE2	Aglianico bianco, Coda di volpe bianca, Coglionara, Mennavacca, Merlot, Piedirosso, Pizzutello bianco, Royal, Roviello, Magliocco, Montonico, Sirica
	142	ERE2	Aglianico bianco, Coda di volpe bianca, Coglionara, Mennavacca, Merlot, Piedirosso, Pizzutello bianco, Royal
CCMP2	207	ERE1	Highly typical of *V*. *vinifera*

Allelic variants in modern cultivars are in accordance with Villano et al. [34].

### Biometric analysis

**Fig 4:**
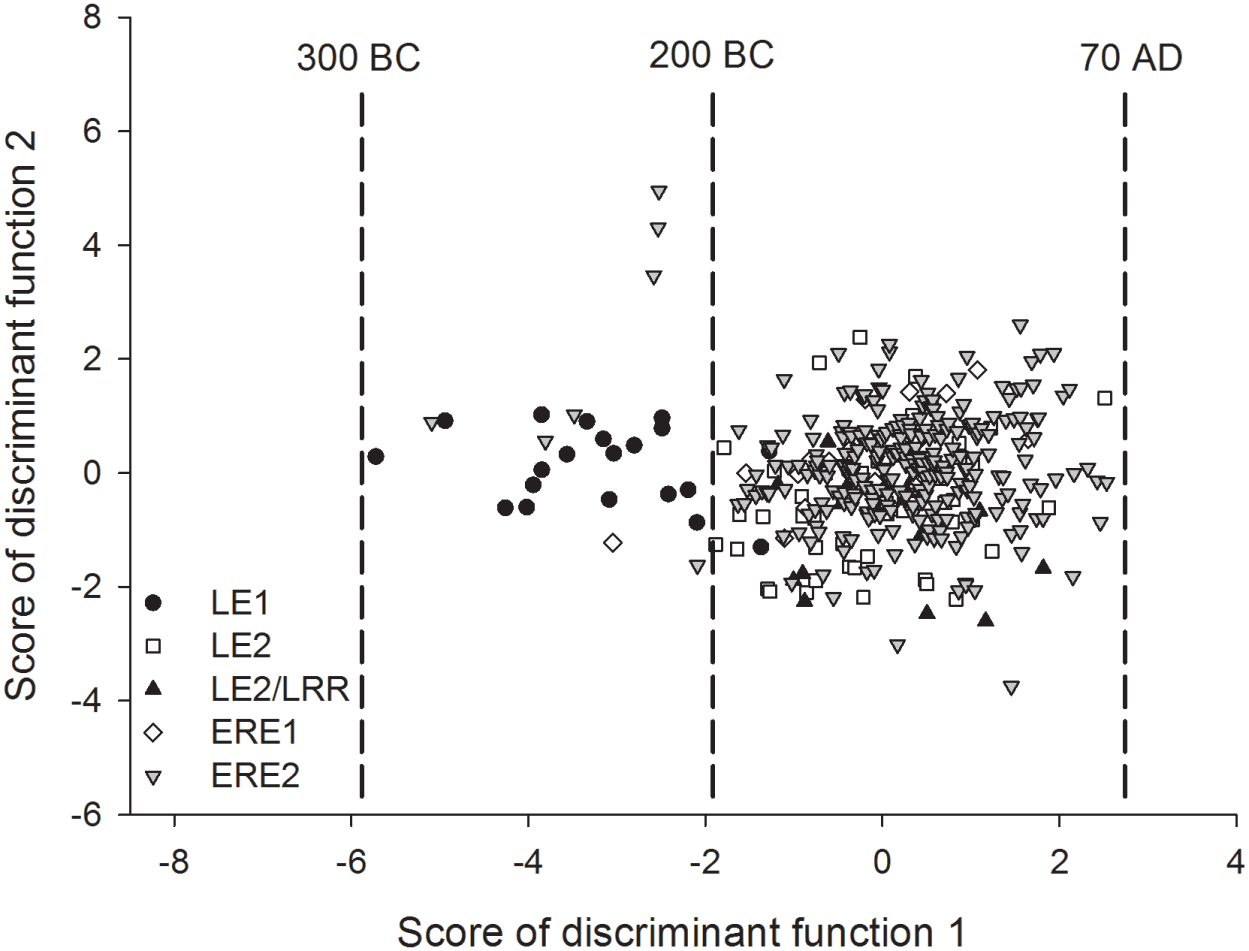
Scatter plot of the scores of discriminant functions 1 and 2 extracted by discriminant analysis for grape pips belonging to five cultural phases. Additional information about these functions is reported in Tables 5 and 6. Each point represents a single pip. Vertical dashed lines are reported to help allocate the pips to different time periods.

The analysis of variance indicated that pips of the LE1 phase had, on average, a lower pip perimeter (15.5 mm), pip area (11.3 mm^2^), pip breadth (3.3 mm), pip length (5.1 mm), stalk length (1.3 mm) and chalaza position (3.4 mm) compared to those of the other four phases (Table 4). The Stummer index did not vary among cultural periods, ranging between 59 and 64 (Table 4). This shape index suggests that, on average, the pips cannot be classified either as cultivated (Stummer index between 44 and 53) or as wild subspecies (Stummer index between 76 and 83). The discriminant analysis extracted four functions, of which only the first two were significant and accounted for 82.1% and 13.7% of total variance, respectively (Table 5). The standardized coefficients of these functions are reported in Table 5. The scores of discriminant function 1 were positively correlated mainly to pip perimeter, area, breath, length, and stalk length, whereas the score of discriminant function 2 was mostly correlated to chalaza position and pip length (Table 6). The discriminant procedures were validated by classification statistics: 43% of pips were correctly allocated to the five original groups previously used for the discrimination. Ninety percent of pips belonging to phase LE1 were correctly reclassified. Discriminant function 1 clearly discriminated 18 out of the 20 pips of LE1 from most of the pips from other phases due to their lower scores (in Fig 4 see the black circle point within the closed curved line). This result suggests that these 18 pips of phase LE1 were characterized at the same time by lower pip perimeter, area, breath, length, and stalk length compared to the samples from the other phases.

**Table 4:** Average morphometric measurements grouped by cultural phase.

Phase code	Number of pips	Pip perimeter (mm)	Pip area (mm2)	Pip breadth (mm)	Pip length (mm)	Stalk length (mm)	Chalaza position (mm)	Stummer index
ERE2	280	19.4a	18.3a	4.2a	6.6a	1.7a	3.9a	63.8
ERE1	23	19.3a	17.0a	4.0ab	6.4a	1.7a	3.8a	63.1
LE2/LRR	16	18.8a	17.2a	3.9b	6.7a	1.6a	3.8a	58.9
LE2	58	19.1a	17.4a	4.0ab	6.5a	1.6a	3.8a	62.4
LE1	20	15.5b	11.3b	3.3c	5.1b	1.3b	3.4b	63.5

**Table 5:** Eigenvalue, percentage and cumulative percentage of explained variance and standardized coefficient of the discriminant functions calculated by discriminant analysis.

Discriminant function	Eigenvalue	Percentage of variance		Standardized coefficients					
		%	Cum. %	Pip perimeter	Pip area	Pip breadth	Pip length	Stalk length	Chalaza position
1	0.566*	82.1	82.1	-0.671	-0.541	1.082	1.132	-0.089	0.490
2	0.094*	13.7	95.7	0.273	1.634	-0.460	-2.095	1.217	-0.119
3	0.022n.s.	3.2	98.9	1.342	-0.488	-0.369	-0.998	0.044	0.656
4	0.007n.s.	1.1	100	-0.635	-1.199	0.473	1.283	0.613	-0.116

* = significant at p < 0.05;

n.s. = not significant

**Table 6:** Correlation coefficient between original variables and the scores of the discriminant functions obtained by discriminant analysis.

Discriminant function	Original variables					
	Pip perimeter	Pip area	Pip breadth	Pip length	Stalk length	Chalaza position
1	0.77	0.72	0.69	0.67	0.61	0.33
2	0.19	-0.12	-0.13	0.40	0.12	0.45
3	-0.24	0.53	-0.06	-0.08	0.32	0.16
4	-0.26	0.07	0.33	-0.47	-0.03	0.76

## Discussion

After due analysis of the plant material in this study we were able to propose new hypotheses on the evolution of viticulture from 300 BC to 100 AD in a well-defined restricted area in the core of the Chianti hills, one of the most important wine grape growing regions in the world. Molecular investigation of 15 pips recovered 14 microsatellite loci, demonstrating good DNA preservation in anoxic contexts. Similar results were also reported for other waterlogged grape pips and woods [1,9,18,40,41]. Having obtained microsatellite data, we first sought to ascertain whether ancient pips found within each layer originated from a single grapevine individual. For ERE2 samples we amplified eight loci and found several SSR polymorphisms among them, supporting the hypothesis that the pips originated from different grapevine individuals. Hierarchical cluster analysis based on genetic distance of the analyzed samples also showed that they do not group according to the corresponding cultural periods. Tentative comparison of the genetic profiles of our ancient samples with modern varieties revealed that several ancient allelic variants are still conserved. This means that they have been preserved during evolution and highlights the possibility of exploiting grape SSR databases to assist domestication and cultivation studies of this important crop. Although several different modern grapevine accessions presented the ancient allelic variants, full correspondence between "ancient" and "modern" genetic profiles was not found. This was to be expected, as also reported elsewhere [1,9,18,42,43], since it would be beyond the bounds of possibility that an ancient specimen could transmit its genetic pattern intact to modern cultivars, especially in SSR regions, which are known to evolve continuously [44].

All available 454 pips were subjected to morphogeometric analysis. Seed morphology has often been used to trace the origin and spread of grapevine domestication and cultivation and to distinguish wild from cultivated grapes [15,42]. Changes in seed morphology have also been reported as indicators of the strength of selection pressure [16]. We found that pip morphology was subject to considerable variation between and within cultural periods. Much of the variability was related to a change in pip size. Most of the pips from the LE1 period tended to be smaller than those in later cultural periods. In addition, LE1 pips were characterized by a shorter stalk (Table 4 and Fig 4). However, the six SSR loci which amplified in all samples across the historical periods did not highlight any clear diachronic evolution of the cultivated genotype (Fig 3). Interestingly, DNA of LE1 samples (300–200 BC) appeared to be relatively similar to that of ERE1 samples (30 BC-40 AD; Fig 3), whereas these two populations of pips differed considerably morphologically (Fig 4).

Thus, integrating the results of the biometric and molecular analyses, we propose that the sudden increase in pip size that occurred starting from early LE2 (around 200 BC) was caused by a change in vineyard management rather than by the selection and introduction of new varieties. Indeed, the classical authors (e.g. Pliny the Elder in Naturalis Historia, book 17, chapter 35) reported that Etruscan viticulture did not include grafting or regular pruning [45]. In addition, vines cultivated by Etruscans were large plants trained up tall live trees or tutors. Such minimally pruned vines have a large number of buds per vine and hence a high crop load. A large number of bunches per vine is known to have negative effects on berry and wine composition [46]. Indeed, Etruscan wine is reported to have been of low quality by several Greek and Roman historians [45]. The Romans had little viticultural knowledge of their own before they came into contact with the Greek and Phoenician wine cultures (3^rd^ century BC) in Magna Graecia (Southern Italy) and in northern Africa [47], respectively. Therefore, the Romans developed a new model of viticulture based on the Etruscan training system (large minimally pruned vines climbing on living trees) [45,48], improved by introducing elements of innovation from Graeco-Phoenician viticulture, like (a) planting vines in regular rows, (b) the use of grafting, and (c) the regular use of pruning [45].

The introduction of regular pruning probably represented one of the most important innovations in Roman viticulture. Pruning is the most effective practice to regulate vine vegetative and reproductive growth [37,49]. It has been amply shown that unpruned or minimally pruned vines tend to have larger fruit yields compared to vines exposed to more intense pruning, and that this leads to a decrease in berry growth [50] and in pip size [51]. Hardie and Aggenbach [51] provide evidence that major changes in vine training, pruning and canopy management significantly affect seed development. Therefore it may be hypothesized that the increase in pip size we measured at around 200 BC was partially due to the introduction of novel canopy management practices that were previously unknown. This occurred 150 years before the definitive end of Etruscan culture in Cetamura (beginning of ERE1 period). Our data thus support the hypothesis that the beginning of modern viticulture in Chianti can be dated around 200 BC, when Cetamura was still Etruscan, but it was closely surrounded by territories that were already under strong Roman influence [52]. Furthermore, the archaeological evidence indicates that in this period: a) on Roman farms (*villae*) there was a significant increase in the presence of wine-making tools (grape presses, vats and jars) [53,54] and b) the Roman amphoras for transporting wine (Dressel 1 and Lamboglia 2 types) replaced those of Massalia (Marseilles) and Graeco-Italic origin, produced respectively in southern France and central-southern Italy [55–59]. In the same period, a similar transition in the type of amphoras adopted occurred in Cetamura [31]. Such evidence of increased interest in enology was the consequence of specific entrepreneurial choices taken by Roman landowners, who became more interested in investing in grape cultivation, wine production and trade after the conquest of most of the Central-Western part of the Mediterranean [52].

Our data suggest that this wine-making revolution in the Italian peninsula also involved the most peripheral and remote areas of the Roman Republic such as Chianti. In Cetamura a change in the viticultural model was required to keep pace with this new trend in wine production. To the best of our knowledge, this is the first study providing scientific evidence to date the precise historical period when significant improvements were introduced in the cultivation model of grapevines in ancient times. Our evidence was obtained by adopting an innovative integrated approach including morphological, molecular and archaeobotanical analyses. Indeed, in previous studies, the morphological analysis of archeological pips was exclusively used to distinguish wild from cultivated subspecies [10–12,16] or, more recently, to compare well-preserved archaeological material with modern grapevine varieties [8,14,15]. Bacilieri et al. [18] suggested that the combined use of molecular markers and morphogeometry is a promising strategy for deciphering the intricate history of grapevine domestication. In our opinion, the potential use of this approach can go well beyond this. Molecular and biometric analyses, especially if interpreted within archaeological and viticultural contexts, can play a major role in future studies to clarify the diversification of grape cultivation and wine making and to track the introduction of technological innovations.

## Conclusions

In this research, the multidisciplinary approach employed to study the waterlogged grapevine remains found in the Chianti area allowed us to detect a sudden change in pip size between the Etruscan and Roman periods. This morphological variation could not be explained by a change in the cultivated variety. Our data suggest that a sudden change in vineyard management strategies may well have occurred in Chianti due to the impact of Roman culture in the 2^nd^ century BC. In this period, there was increasing Roman interest in investing in grape cultivation and wine making to satisfy the growing demand after the conquest of the Central-Western Mediterranean. The introduction of innovative vineyard management practices that were unknown to the Etruscans (such as planting vines in regular rows, the use of grafting, and the regular use of pruning) may have induced vine physiological conditions that were more favorable for pip growth. Our study dates the sudden impact of the Romans on viticulture at around 200 BC, providing new scientific support for the classical authors who maintained that Etruscan viticulture was “primitive” and allowed the production of low-quality wines. Therefore, the Chianti area represents an exemplary case of how and when Roman culture contributed to developing modern viticulture prior to the spread of such influence across large parts of Europe.

## Supporting information

S1 Methods Detailed methodology adopted for molecular analysis. Ancient DNA extraction, PCR, capillary electrophoresis and data analysis procedures are reported.
(DOCX)

S1 Table Microsatellite markers used for archaeological grape pips genotyping. Locus name, primer sequences (5’–3’), tested annealing temperatures (Ta) and references are reported. The asterisked loci amplified in aDNA analysis.
(DOCX)
