# Supplementary material for: Dating the beginning of the Roman viticultural model in the Western Mediterranean: The case study of Chianti (Central Italy)
Source: PLoS One. 2017 Nov 15;12(11):e0186298. doi: 10.1371/journal.pone.0186298 (PMC5687709; doi:10.1371/journal.pone.0186298)
Supplement: S1 Methods — Ancient DNA extraction, PCR, capillary electrophoresis and data analysis procedures are reported. (DOCX) [file pone.0186298.s001.docx]

**S1 Methods. Detailed methodology adopted for molecular analysis.**

Ancient DNA extraction, PCR, capillary electrophoresis and data analysis procedures are reported.

The experimental procedures described in this work were conducted in a physically separated workplace dedicated to aDNA, which has never been used for isolation of contemporary grapevine nucleic acids. The laboratory is provided of a contaminant resistant service made up of UV lights, HEPA filters and a system that continuously generates a positive pressure. The workplace was sterilized thoroughly with a 50% (v/v) sodium hypochlorite solution (6% active chlorine) for 10 min and rinsed with sterile distilled water. All the instruments and the material employed during this study were previously sterilized by autoclave and/or UV-light and/or sodium hypochlorite. Twenty-two ancient grape pips belonging to different phases were used for DNA extraction. Due to the limited number of samples excavated from the same layer, a single-seed extraction was carried out in order to capture the genetic signature of each sample rather than mixed signal from multiple individuals. To remove external contaminant sources of DNA, pips were briefly washed in EtOH 70%, subsequently in a dilute bleach solution (10% of the commercial strength), and then rinsed in analytical grade H2O. Pips were dried naturally under a laminar flow hood before being manually crushed with liquid nitrogen. Extraction of aDNA was conducted under a laminar flow hood to avoid any contamination. DNA from ancient grape pips was extracted using the ChargeSwitch Forensic DNA Purification Kit (Life Technologies, Carlsbad, CA, USA). Positive controls were avoided to circumvent contamination risk, while negative ones were always performed. Extracted DNA was diluted to a concentration of 0.1 ng/uL and stored in Eppendorf at -20°C. Microsatellite analysis was carried out with 23 nuclear and 11 chloroplast markers as reported in S1 Table. Among them, we included the nine SSR loci (VVS2, VVMD5, VVMD7, VVMD25, VVMD27, VVMD28, VVMD32, VrZAG62, and VrZAG79) that the European scientific community selected and chose for grapevine identification, standardization and exchange of information^[[1]](#endnote-1)^. All the PCR amplifications were repeated at least three times using different thermocyclers situated in separated laboratories where different research teams work. PCR reaction were carried out as reported in Villano et al.^[[2]](#endnote-2)^ with the following modifications: Platinum Taq “HiFi” (1 U) (Life Technologies) and BSA were used in the reaction. Cycling conditions consisted of 94°C for 2 min; 35 cycles of 30s at 94°C, 30s at specific Ta, 1 min at 72 °C, followed by 30 min at 72 °C. Amplification products were checked and quantified on 2% agarose/TAE gel using 1Kb plus ladder (Life Technologies). Amplicons were separated with the ABI PRISM® 3130 DNA Analyzer system (Life Technologies). Size calibration was performed with the molecular weight ladder GenScan® 500 ROXTM Size Standard (Life Technologies). Electropherograms were then analyzed using the software Peak scanner ver. 1.0 (Applied Biosystems, Foster City, CA, USA). Because the chloroplast genome is uniparentally inherited and thus not recombining, it was treated as one locus, and the different haplotypes were treated as alleles. SSR profiles were compared with the database owned by our group, consisting of 62 grapevines SSR profiles previously obtained^[[3]](#endnote-3)^ and lastly integrated with additional 60 profiles from as many genotypes. For markers which gave amlicons in at least one pip per phase, data were scored for the presence or absence of each allele in all genotypes, and a genetic distance matrix was calculated using the Dice coefficient^[[4]](#endnote-4),^^[[5]](#endnote-5)^. A dendrogram was built through the UPGMA (Unweighted Pair Group Method with Arithmetic Mean) method using R software, version 3.2.1 (2015-06-18).

1. This P, Jung A, Boccacci P, Borrego J, Botta R, Costantini L, et al. Development of a standard set of microsatellite reference alleles for identification of grape cultivars. Theor Appl Genet. 2004;109: 1448-1458. doi: 10.1007/s00122-004-1760-3 [↑](#endnote-ref-1)
2. Villano C, Carputo D, Frusciante L, Santoro X, Aversano R. Use of SSR and retrotransposon-based markers to interpret the population structure of native grapevines from Southern Italy. Mol Biotechnol. 2014;56: 1011-20. doi: 10.1007/s12033-014-9780-y [↑](#endnote-ref-2)
3. [↑](#endnote-ref-3)
4. Dice LR. Measurement of the amount of ecological association between species. Ecology. 1945;26: 297–302. doi: 10.2307/1932409 [↑](#endnote-ref-4)
5. Sneath PHA, Sokal RR. Numerical taxonomy- the principles and practice of numerical classification. San Francisco: Freeman; 1973. [↑](#endnote-ref-5)
