# Supplementary material for: Dating the beginning of the Roman viticultural model in the Western Mediterranean: The case study of Chianti (Central Italy)
Source: PLoS One. 2017 Nov 15;12(11):e0186298. doi: 10.1371/journal.pone.0186298 (PMC5687709; doi:10.1371/journal.pone.0186298)
Supplement: S1 Table — Locus name, primer sequences (5’–3’), tested annealing temperatures (Ta) and references are reported. The asterisked loci amplified in aDNA analysis. (DOCX) [file pone.0186298.s002.docx]

**S1 Table**. **Microsatellite markers used for archaeological grape pips genotyping.**

Locus name, primer sequences (5’ - 3’), tested annealing temperatures (Ta) and references are reported. The asterisked loci amplified in aDNA analysis.

| Locus | Forward primer | Reverse primer | Reference |
| --- | --- | --- | --- |
| *Nuclear* |  |  |  |
| VrZAG29 | ataaccaggacaaagttattcaagcc | acccaattgaccatcttttatgctg | Sefc et al.,1999 |
| VVIp60 | ggggaataactaaattgaggat | gtatgaatgcggatagtttgtg | Merdinoglu et al.,2005 |
| VVIb01 | tgaccctcgaccttaaaatctt | tggtgagtgcaatgatagtaga | Merdinoglu et al.,2005 |
| VrZAG12 | ctgcaaataaatattaaaaaattcg | aaatcctcggtctctagccaaaagg | Sefc et al.,1999 |
| VrZAG47* | ggtctgaatacatccgtaagtatat | acggtgtgctctcattgtcattgac | Sefc et al.,1999 |
| VrZAG21 | tcattcactcactgcattcatcggc | ggggctactccaaagtcagttcttg | Sefc et al.,1999 |
| VrZAG7 | gtggtagtgggtgtgaacggagtgg | aacagcatgacatccacctcaacgg | Sefc et al.,1999 |
| VrZAG79 | agattgtggaggagggaacaaaccg | tgcccccattttcaaactcccttcc | Sefc et al.,1999 |
| VVMD27 | gtaccagatctgaatacatccgtaagt | acgggtatagagcaaacggtgt | Bowers et al., 1999 |
| VVMD28 | aacaattcaatgaaaagagagagagaga | tcatcaatttcgtatctctatttgctg | Bowers et al., 1999 |
| VVMD32 | tatgattttttaggggggtgagg | ggaaagatgggatgactcgc | Bowers et al., 1999 |
| VVS5* | attgatttatcaaacaccttctacat | tagaaagatggaaggaatggtgat | Thomas et al.,1993 |
| VrZAG62 | ggtgaaatgggcaccgaacacacgc | ccatgtctctcctcagcttctcagc | Sefc et al.,1999 |
| VVS4 | ccatcagtgataaaacctaatgcc | cccaccttgcccttagatgtta | Thomas et al.,1993 |
| VVIq52 | taaaaggatggtagatgacaga | acaggaaagtgttcaatggtta | Merdinoglu et al.,2005 |
| VVIC05 | aagcaagttgaagaacgtgtaagtc | ggcgaagaatcttactgagaattg | Merdinoglu et al.,2005 |
| VVMD25 | ttccgttaaagcaaaagaaaaagg | ttggatttgaaatttattgagggg | Bowers et al., 1999 |
| VVS2* | cagcccgtaaatgtatccatc | aaattcaaaattctaattcaactgg | Thomas et al.,1993 |
| VVIh54 | ccgcacttgtgttgaatttcag | caaaccgtttttacaccagcag | Merdinoglu et al.,2005 |
| VrZAG112* | cgtttaaagccagctgaatcttggg | tggctccatactgcttcacgtaggc | Sefc et al.,1999 |
| VVMD5 | ctagagctacgccaatccaa | tataccaaaaatcatattcctaaa | Bowers et al., 1999 |
| VVMD7* | agagttgcggagaacaggat | cgaaccttcacacgcttgat | Bowers et al., 1999 |
| VVIn16 | acctctataagatcctaacctg | aagggagtgtgactgatatttc | Merdinoglu et al.,2005 |
| *Chloroplast* |  |  |  |
| CCMP1* | caggtaaacttctcaacgga | ccgaagtcaaaagagcgatt | Weising et al.,1997 |
| CCMP2* | gatcccggacgtaatcctg | atcgtaccgagggttcgaat | Weising et al.,1997 |
| CCMP3* | cagaccaaaagctgacatag | gtttcattcggctcctttat | Weising et al.,1997 |
| CCMP4 | aatgctgaatcgaygaccta | ccaaaatattbggaggactct | Weising et al.,1997 |
| CCMP5* | tgttccaatatcttcttgtcattt | aggttccatcggaacaattat | Weising et al.,1997 |
| CCMP6* | cgatgcatatgtagaaagcc | cattacgtgcgactatctcc | Weising et al.,1997 |
| CCMP7* | caacatataccactgtcaag | acatcattattgtatactctttc | Weising et al.,1997 |
| CCMP8* | ttggctactctaaccttccc | ttctttcttatttcgcagdgaa | Weising et al.,1997 |
| CCMP10 | tttttttttagtgaacgtgtca | ttcgtcgdcgtagtaaatag | Weising et al.,1997 |
| ccSSR5* | tctgataaaaaacgagcagttct | gagaaggttccatcggaacaa | Chung et al.,2003 |
| ccSSR14* | gggtataatggtagatgccc | gccgtagtaaataggagagaaa | Chung et al.,2003 |

Chung SM, Staub JE. The development and evaluation of consensus chloroplast primer pairs that possess highly variable sequence regions in a diverse array of plant taxa. Theor Appl Genet. 2003;107: 757–767. doi: 10.1007/s00122-003-1311-3

Sefc KM, Regner F, Turetschek E, Glössl J, Steinkellner H. Identification of microsatellite sequences in *Vitis riparia* and their applicability for genotyping of different *Vitis* species. Genome. 1999;42**:** 367–373.

Merdinoglu D, Butterlin G, Bevilacqua L, Chiquet V, Adam-Blondon AF, Decroocq S. Development and characterization of a large set of microsatellite markers in grapevine (*Vitis vinifera* L.) suitable for multiplex PCR. Mol Breeding. 2005;15: 349–366. doi: 10.1007/s11032-004-7651-0

Bowers JE, Dangl GS, Meredith CP. Development and Characterization of Additional Microsatellite DNA Markers for Grape. Am J Enol Vitic. 1999;50: 243–246.

Thomas MR, Scott NS. Microsatellite repeats in grapevine reveal DNA polymorphisms when analysed as sequence-tagged sites (STSs). Theor Appl Genet. 1993;86: 985–990. doi: 10.1007/BF00211051

Weising K, Winter P, Hüttel B, Kahl G. Microsatellite Markers for Molecular Breeding. J Crop Prod. 1997;1: 113–143. doi: 10.1300/J144v01n01_06
